# Inducible nitric oxide synthase mediates DNA double strand breaks in Human T-Cell Leukemia Virus Type 1-induced leukemia/lymphoma

**DOI:** 10.1186/s12977-015-0196-y

**Published:** 2015-08-12

**Authors:** Hicham H Baydoun, Mathew A Cherian, Patrick Green, Lee Ratner

**Affiliations:** Division of Molecular Oncology, Department of Medicine Campus, Washington University School of Medicine, 660 South Euclid Avenue, St Louis, MO 63110 USA; Department of Molecular Microbiology, Washington University School of Medicine, St Louis, MO USA; Department of Veterinary Biosciences, The Ohio State University, Columbus, OH USA

**Keywords:** ATLL, HTLV-1, Tax, Nitric oxide, NF-κB, DNA damage, DSBs

## Abstract

**Background:**

Adult T-cell leukemia/lymphoma (ATLL) is an aggressive and fatal malignancy of CD4^+^ T-lymphocytes infected by the Human T-Cell Virus Type 1 (HTLV-1). The molecular mechanisms of transformation in ATLL have not been fully elucidated. However, genomic instability and cumulative DNA damage during the long period of latency is believed to be essential for HTLV-1 induced leukemogenesis. In addition, constitutive activation of the NF-κB pathway was found to be a critical determinant for transformation. Whether a connection exists between NF-κB activation and accumulation of DNA damage is not clear. We recently found that the HTLV-1 viral oncoprotein, Tax, the activator of the NF-κB pathway, induces DNA double strand breaks (DSBs).

**Results:**

Here, we investigated whether any of the NF-κB target genes are critical in inducing DSBs. Of note, we found that inducible nitric oxide synthase (iNOS) that catalyzes the production of nitric oxide (NO) in macrophages, neutrophils and T-cells is over expressed in HTLV-1 infected and Tax-expressing cells. Interestingly, we show that in HTLV-1 infected cells, iNOS expression is Tax-dependent and specifically requires the activation of the classical NF-κB and JAK/STAT pathways. A dramatic reduction of DSBs was observed when NO production was inhibited, indicating that Tax induces DSBs through the activation of NO synthesis.

**Conclusions:**

Determination of the impact of NO on HTLV-1-induced leukemogenesis opens a new area for treatment or prevention of ATLL and perhaps other cancers in which NO is produced.

## Background

Human T-Cell Leukemia Virus Type 1 (HTLV-1) is the etiological agent of adult T-cell leukemia-lymphoma (ATLL), an aggressive and fatal malignancy of CD4^+^ T-lymphocytes, for which an effective treatment is not yet available. HTLV-I is the only transmissible retrovirus that causes cancer in humans. HTLV-1 can be transmitted sexually, by intravenous drug abuse or vertically through breast-feeding. Approximately, twenty million individuals worldwide are infected with HTLV-1 [1], and 2–5% of infected individuals develop ATLL after a long period of latency, typically forty or more years. It is widely believed that during the period of latency a number of oncogenic events accumulate and lead to the transformation of infected lymphocytes [2, 3].

The oncogenic events leading to ATLL are largely unknown and are under active investigation. Although several HTLV-1 viral proteins play a role in viral pathogenesis, the HTLV-1 encoded oncoprotein Tax provides the major contribution to the leukemogenic process [4]. Tax perturbs multiple T-cell proliferation pathways, including the NF-κB, JAK/STAT, PI3K/AKT and TGF-β pathways [5–11]. Tax also inhibits tumor suppressors, induces genomic instability and activates angiogenesis. Furthermore, Tax expressing T-cells, which are able to induce tumors in vivo and to form colonies in vitro [11–13], reveal a massive alteration in gene expression, suggesting that Tax regulates a large number of genes. Yet, the genes directly affected by Tax have not been completely characterized [14, 15].

Nitric oxide (NO) is an important cellular signaling molecule involved in many physiological and pathological processes. It is generated from l-arginine by the various isoforms of nitric oxide synthase (NOS). Constitutively expressed endothelial NOS (eNOS) and neuronal NOS (nNOS) synthesize NO and are involved in vasodilatation and neuronal communication, respectively. However, inducible NOS (iNOS) is synthesized de novo in response to a variety of inflammatory mediators [16]. Cytokines and chemokines activate the expression of iNOS, which catalyzes the production of large amounts of NO in activated T-cells, and macrophages. Inducible NO is the precursor of the highly reactive nitrogen species peroxynitrite (ONOO^−^), an obligatory factor in oxidative DNA damage. NO also induces nitrosative stress, apoptosis, mitochondrial damage, cytostasis, and cytolysis [17, 18]. However when produced at low concentrations by macrophages or activated T-cells during chronic inflammation, it promotes tumor cell proliferation, migration, invasion, and resistance to apoptosis [19]. Thus, inducible NO belongs to a category of molecules that imposes a critical balance between pro and anti-apoptotic mechanisms during cancer development. To date, drugs targeting NO abundance in mouse models of colon, breast and ovarian cancers dramatically decreased tumor progression [20–25].

We recently showed that the HTLV-1 Tax protein induces DNA double strand breaks (DSBs) [26], the most detrimental form of genetic damage, due to inhibition of transcription, replication and chromosome segregation. In the present study, we found that HTLV-1 infected T-cells and Tax expressing cells exhibit high levels of intracellular NO. Since NO, and its product peroxynitrite, are highly reactive molecules that inflict DNA damage, we hypothesized that the HTLV-1 Tax protein induces DSBs through the induction of NO synthesis. Here we demonstrate that Tax specifically induces DSBs through the expression of iNOS via activation of the NF-κB and JAK/STAT pathways. In a recent work, Tax has been shown to impair DNA replication forks and increase DNA breaks. The authors included experiments in which Tax induced breaks by NO through the NF-κB pathway [27]. However, those experiments were carried out only in the epithelial 293T cell line that does not express NOS genes [28, 29]. In addition, a non-selective drug (L-NMMA) was used in that study to inhibit NO release, but more specific drugs to iNOS or genetic knockdown experiments were not utilized. Lastly, those experiments did not include Western blot or immunofluorescence staining for γ-H2AX which are standard evaluations of DNA breaks. By inducing DNA damage, NO could be a key player in the onset and progression of HTLV-1 induced leukemia.

## Results

### HTLV-1 transformed T-cells display high levels of nitric oxide

**Fig. 1:**
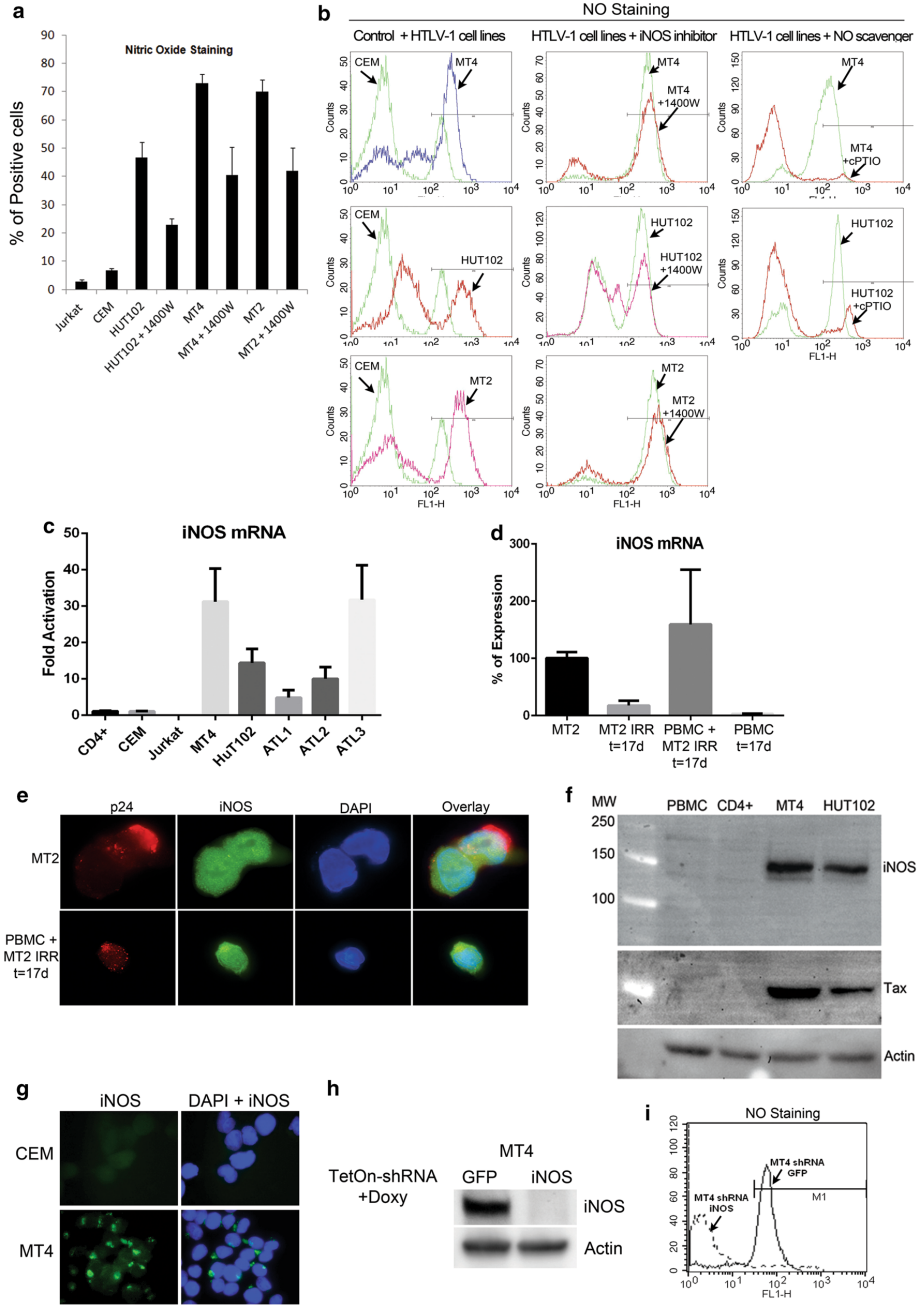
High levels of NO and iNOS are detected in HTLV-1 transformed cell lines and ATLL patients’ samples. **a** HTLV-1 transformed cell lines MT2, MT4, and HuT102, and transformed human T-cell controls Jurkat and CEM treated with DMSO or with 1400 W, an iNOS selective inhibitor, were stained with DAF-2DA to measure the intracellular pool of NO. The histograms represent the average of three different experiments with the indicated standard deviations. **b** Flow cytometry analyses of the intracellular pool of NO shows the difference between control cells and HTLV-1 infected cell lines (*1st column*), HTLV-1 infected cells untreated or treated with iNOS inhibitor, 1400 W (*2nd column*), and HTLV-1 infected cells untreated or treated with NO scavenger, cPTIO (*3rd column*). **c** qRT-PCR of the intracellular pool of iNOS mRNA showed a specific increase in HTLV-1 infected cells and in ATLL patients’ samples but not in the control CD4^+^, Jurkat, and CEM cells. GAPDH was used as an internal control for mRNA quantification. **d** Real-time measurement of iNOS mRNA of freshly infected PBMCs with irradiated MT2 cells. iNOS mRNA pool is shown in irradiated MT2 cells alone, non-irradiated MT2 cells, and in uninfected PBMCs at 17 days of infection. **e** Immunoflurescence staining of Gag protein, p24, and iNOS in MT2 and freshly infected PBMC at 17 days of infection. **f** Western blot analysis of iNOS protein expression shows the expression of iNOS in MT4 and HuT102 cell lines but not in the control PBMC or CD4^+^ primary cells. **g** Immunofluorescence staining of iNOS protein in CEM control cells and in HTLV-1 transformed human MT4 cells. **h** MT4 cells expressing inducible TetOn control shRNA (GFP) or TetOn shRNA directed against iNOS mRNA were established, and iNOS expression was measured by Western blot. **i** NO measurement by DAF-2DA showing that depletion of iNOS expression by shRNA reduces completely the production of NO.

Nitric oxide is a key component of reactive nitrogen intermediates, which constitute major sources of DNA damage during chronic inflammation. They are involved in the initial events of tumorigenesis as well as in inflammatory responses involved in cancer immunity [30]. Our cancer model, HTLV-1 induced leukemia, is a hematopoietic malignancy characterized by constitutive activation of the NF-κB pathway that drives a potent inflammatory response [6, 31]. In order to investigate the mechanisms by which NO affects genomic instability in this model, we first evaluated the concentration of NO in a variety of human HTLV-1 transformed T-cell lines: MT2, MT4, and HuT102. We used the human T-ALL cell lines, Jurkat and CEM, as controls. The staining of the intracellular pool of NO in these cell lines using 4,5-Diaminofluorescein Di-acetate (DAF-2DA) showed that HTLV-1 transformed T-lymphocytes display high levels of intracellular NO when compared to control cell lines (Fig. 1a, b first column). In activated T lymphocytes, the inflammatory response induces the expression of inducible nitric oxide synthase (iNOS) which constitutes the main source of nitric oxide production. Treatment of HTLV-1 infected cells for 48 h with a selective inhibitor of iNOS (1400 W), inhibited NO production by ~40%, indicating that iNOS was at least partially responsible for production of NO in HTLV-1 transformed cell lines (Fig. 1a, b second column). An increase of 1400 W concentration could not potentiate its inhibitory effect, suggesting that NO is still produced in the cells. However, the treatment of HTLV-1 infected cells for 24 h with a NO scavenger, such as cPTIO, reduced the amount of NO by 90% indicating the specificity of NO staining by DAF-2DA (Fig. 1b, third column).

iNOS expression in ATLL cells has previously been reported [32, 33]. We verified these observations, using real time RT-PCR to demonstrate that MT4 and Hut102 cells express significantly higher levels of iNOS mRNA than control CD4^+^, Jurkat and CEM T cells (Fig. 1c). In addition, RNA was extracted from PBMC samples of patients with acute ATLL (patients 1, 2 and 3, Table 1). All three ATLL samples showed activation of iNOS expression when compared to the level detected in control cells (Fig. 1c). To determine if the elevation of iNOS expression in HTLV-1 infected cells and in ATL samples was due to HTLV-1 viral infection, we measured iNOS expression in freshly infected PBMC with HTLV-1 virus. We co-cultured PBMCs from a healthy donor with irradiated MT2 cells (ratio 10–1) for 17 days and measured the expression of iNOS mRNA. After 17 days in culture, irradiated MT2 cells and non-infected PBMC expressed very low levels of iNOS when compared to non-irradiated MT2 cells. However, the freshly infected PBMCs expressed a comparable level of iNOS as that found in non-irradiated MT2 cells (Fig. 1d). In freshly infected PBMCs, we also detected by immunofluorescence the expression of HTLV-1 p24 gag protein as an indication of viral infection (Fig. 1e).

**Table 1:** Patient characteristics

Pt #	ATLL Subtype	Stage	Wbc	CD3	CD4	CD25	LDH	Ca	BM	PVL
1	Acute	IV	135.7	119	119	67	550	14.1	81%	145.4
2	Acute	IV	20.6	6.3	6.4	5.9	1,269	10.1	59%	77.3
3	Acute	IV	69.4	65.2	63.1	nd	1,496	9	nd	88.4

ATLL subtype, Shimoyama classification; BM, bone marrow % malignant cell; Ca, calcium (mg/dL, 8.5–9.9 normal); CD3, CD4 or CD25 (K/cumm); LDH, lactate dehydrogenase (U/L, normal <250); nd, not determined; PVL, proviral load (copies/100 PBMCs); stage, Ann Arbor lymphoma stage; wbc, white blood cell count (K/cumm).

As expected, the protein level of iNOS was increased in HTLV-1 positive MT4 and HuT102 transformed cell lines as compared to control PBMC and CD4^+^ cells (Fig. 1f). Intracellular fluorescence staining was also performed to determine the localization of iNOS. Images presented in Fig. 1g indicate that iNOS is expressed in more than 90% of MT4 cells, but it is absent from control CEM T cells. iNOS was localized in the cytoplasm of MT4 cells in an aggresome distribution, as previously described [34].

To determine if elevated levels of iNOS in MT4 cells were responsible for the high level of NO, we established stable MT4 cell lines that express a TetOn inducible shRNA to downregulate the expression of a control gene (GFP) or that of iNOS. MT4-TetOn GFP shRNA and MT4-TetOn iNOS shRNA cells were induced by doxycycline for 48 h and iNOS protein expression was assessed by Western blot. Figure 1h showed complete depletion of iNOS expression in MT4 cells expressing specific shRNA against iNOS mRNA as compared to MT4 cells expressing control shRNA. Similarly, the production of nitric oxide was completely reduced in MT4-TetOn iNOS shRNA expressing cells, when compared to MT4 expressing control shRNA (Fig. 1i), indicating that iNOS is the major enymatic catalyzer of nitric oxide production in HTLV-1 transformed cell lines. The depletion experiment and the related reduction in NO production also suggested that 1400 W may not be sufficiently active to completely inhibit iNOS activity. Similar results were obtained with less selective drugs such as L-NMMA and L-NIL (data not shown).

### iNOS induces DNA double strand breaks (DSBs)

**Fig. 2:**
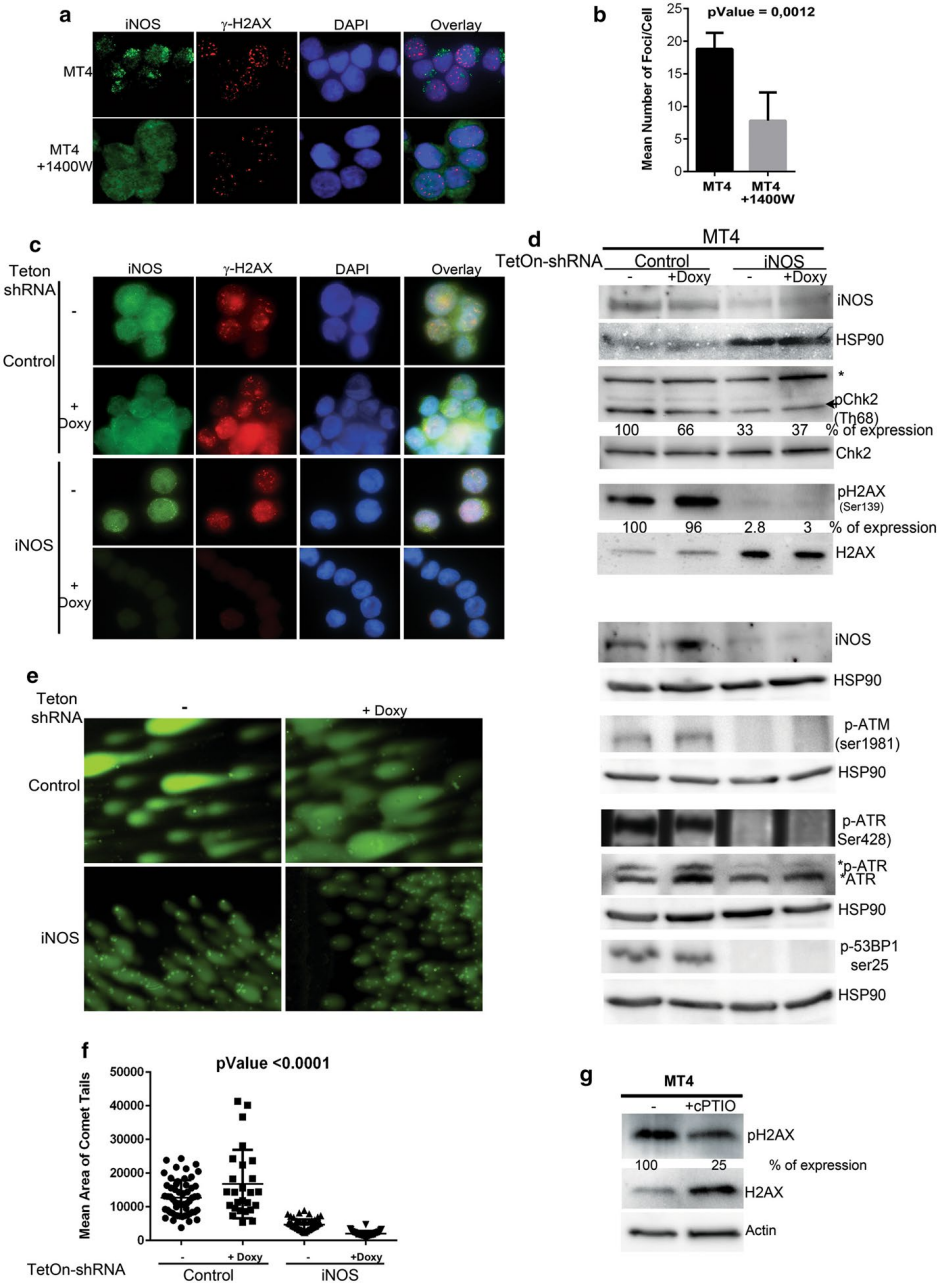
iNOS inhibition is associated with a reduction of the DNA damage response. **a** Double immunofluorescence staining of MT4 cells treated with or without iNOS inhibitor, 1400 W (7 nM), for 48 h, using specific primary antibodies directed against iNOS, and γ-H2AX marker of DSBs and followed by secondary antibodies conjugated to Alexa fluor 488 (*green* for iNOS) and Alexa fluor 596 (*red* for γ-H2AX), respectively. DAPI dye was used to stain the nucleus of the analyzed cells. **b** The histograms represent an accurate estimation of the number of DSBs foci in MT4 cells and MT4 cells treated with iNOS inhibitor. **c** The same double immunofluorescence staining was also carried out on MT4 cells expressing doxycycline inducible TetOn control shRNA or MT4 inducible TetOn shRNA directed against iNOS mRNA. **d** MT4 cells that express inducible TetOn-shRNA control or shRNA specifically targeting the mRNA of iNOS were not induced or induced for 48 h with doxycycline and blotted for iNOS, p-H2AX, H2AX, pChk2, Chk2, p-ATM, p-ATR, p35BP1 and HSP90 as an internal control. Hsp90 blots are shown for each set of separately loaded cell lysates. * non-specific band. **e** Comet alkaline assay method that detects DNA breaks was used for evaluation of DNA damage in the same cells tested above. **f** The object count module was used to measure the area of comet tails (DNA migration), and calculate DNA damage parameters. At least 100 randomly selected cells were analyzed per sample. **g** Western blot on the extracts of MT4 cell untreated or treated with cPTIO (NO scavenger) using p-H2AX and total H2AX antibodies.

Since NO is an obligatory factor in oxidative DNA damage, we wanted to determine whether iNOS induces DSBs through NO synthesis. Induction of DSBs generates a DNA damage response orchestrated by the master regulator ATM (Ataxia Telangiectasia Mutated) that activates a cascade of effectors to repair the DNA defects. ATM-mediated phosphorylation at serine 139 of the histone H2AX protein (γ-H2AX) is the critical step to mark the DNA damage sites and leads to the formation of γH2AX foci [35]. ATM also phosphorylates and activates a set of sensors and effectors involved in DNA repair, which are gradually recruited to γH2AX foci. To determine the effect of iNOS inhibition on the formation of γH2AX foci, we stained iNOS and pH2AX proteins in MT4 cells with specific antibodies (Fig. 2a). In addition, MT4 cells treated for 48 h with the iNOS inhibitor, 1400 W, were also examined by immunofluorescence microscopy. Immunofluorescence images showed that inhibition of iNOS activity was associated with a decrease in the number of DSB foci stained with γH2AX (Fig. 2a, b). To quantify the effects of iNOS inhibitor, the number of DSB foci was measured in 100 cells, and demonstrated a decrease of 60% in MT4 cells treated with iNOS inhibitor when compared to untreated MT4 cells (Fig. 2b). We also examined effects on the number of γH2AX foci in MT4 expressing shRNA against iNOS mRNA by the TetOn inducible system. Expression of the iNOS shRNA markedly reduced the number of γH2AX foci (Fig. 2c, last row). However, the expression of control shRNA in MT4 cells had no effect on the number of γH2AX foci (Fig. 2c).

The DNA damage response was also assessed by Western blot using specific antibodies that recognize different phospho-proteins of the DNA damage response, including ATM, ATR (ataxia telangiectasia and Rad3-related protein), 53BP1 (p53 binding protein 1), the cell cycle delay checkpoint protein Chk2, and p-H2AX. The blot results were consistent with those obtained with fluorescent microscopy and showed a reduction of the DNA damage response in MT4 cells in which iNOS was almost completely depleted by shRNA. MT4 cells expressing control shRNA retained a high level of pATM, pATR, p53BP1, pH2AX, and pChk2 (Fig. 2d). Of note, the western blot results showed that iNOS expression was also depleted in MT4 cells in which the TetOn system expressing shRNA against iNOS mRNA was not induced by doxycycline. This observation was due to the leakiness of the TetOn system and the fact that MT4 cells were not maintained in tetracycline-free media to keep the TetOn system functional. Consistently, the reduction of the DNA damage response correlated with the decrease in iNOS expression. The mono and poly-ubiquitinated forms of H2AX that play a critical role in H2AX Ser-139 phosphorylation (γH2AX), and facilitate the recruitment of other factors to DNA damage foci [36] were detected in MT4 cells expressing control shRNA but not in MT4 cells expressing iNOS shRNA (data not shown). The correlation between iNOS depletion and the decrease of the DNA damage response was also observed in HuT102 cells (data not shown), another HTLV-1 transformed cell line, indicating that DSB induction by iNOS is specific, and is not cell line dependent. Taken together, these results indicate that inducible NO synthesis constitutes an important source of DNA damage in HTLV-1 infected cells. Neither inhibition of iNOS activity, or depletion of iNOS expression had an effect on the viability of cells (data not shown) as measured by Annexin V and propidium iodide staining, suggesting that apoptosis was not affected under those conditions.

For functional evaluation of the effect of iNOS inhibition on the induction of DNA damage in HTLV-1 transformed cells, we used the comet assay, alkaline method, which is a single cell electrophoresis technique that detects both single-strand and double-strand breaks. NO and its reactive product (ONOO^−^) induce DNA modifications that start as single-strand breaks (SSB) which then lead to the formation of double-strand breaks (DSB). The comet tail was scored according to DNA content, by measuring integrated fluorescence intensity. We used the object count module that is available in the microscope NIS elements software to measure the length, area, and intensity of tails (DNA migration), and calculated DNA damage parameters (Fig. 2e, f). At least 100 randomly selected cells were analyzed per sample. As expected, MT4 cells expressing control shRNA, which retain high levels of iNOS migrated with relatively long tails (Fig. 2e, first panel and f). The depletion of iNOS expression by the TetOn-shRNA system showed a reduction in tail area of comets (Fig. 2e, second panel and f). Likewise, treatment of MT4 with NO scavenger, cPTIO, for 48 h reduced p-H2AX expression by 75% confirming that NO is the major source of damage to DNA in the HTLV-1 infected cells (Fig. 2g).

### HTLV-1 Tax protein activates the expression of iNOS

**Fig. 3:**
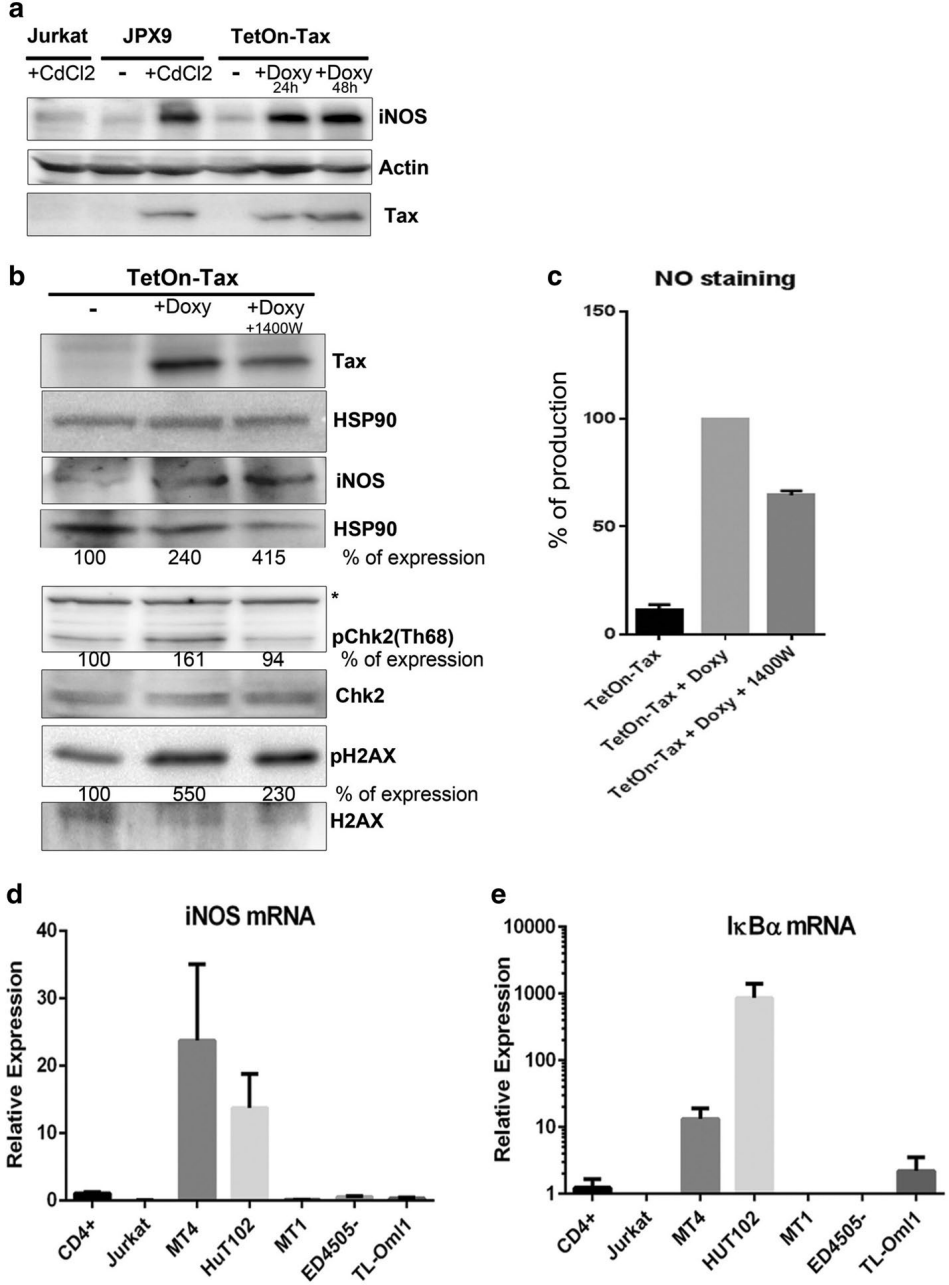
HTLV-1 Tax specifically induces the activation of iNOS expression. **a** Tax inducible cell lines JPX9 and TetOn-Tax were induced for 48 h with CdCl_2_ (40 μM) and doxycycline (500 ng/ml) for 24 and 48 h respectively, to express the Tax protein. Cell extracts were subject to Western blot analysis using antibodies against iNOS, Tax and actin. **b** Doxycyline-induced TetOn-Tax cells were left untreated or treated with 1400 W (7 nM) for 48 h. Western blots were performed on the cell extracts by using anti-p-H2AX, anti-pChk2, anti-H2AX, anti-Chk2, and anti-iNOS antibodies. The percentage of expression of DNA damage response after iNOS inhibition was quantified by the ratio of phosphoprotein on the total amounts of proteins, and it was indicated underneath the Western blot. * non-specific band. **c** NO staining with DAF-2DA was performed on two experiments in which TetOn-Tax was induced and cells treated with iNOS inhibitor, 1400 W, to show that the reduction in DNA damage response was associated with a relative decrease in NO production. **d**, **e** Real time PCR of iNOS and IκBα mRNA in negative control CD4^+^ and Jurkat cells, in HTLV-1 transformed cell lines MT4 and HuT102, and in three Tax-negative ATL cell lines, MT1, ED4505- and TL-Oml1.

Since HTLV-1 Tax is an activator of T-cell proliferation pathways, we wanted to determine whether Tax was involved in the activation of iNOS expression. We used JPX9 and TetOn-Tax human Jurkat T-cell lines that contain stable inducible systems for the expression of Tax with CdCl_2_ [37] and doxycycline [38], respectively. Tax was induced for 24 h or 48 h, and Western blot was performed using anti-iNOS and anti-Tax specific antibodies. The results showed a specific increase of iNOS expression when Tax was induced (Fig. 3a). These findings indicate a dose-dependent correlation between Tax and iNOS expression. Of note, the reagents used to induce Tax expression had no effect on iNOS expression since Jurkat control cells treated with CdCl_2_ did not show iNOS expression (Fig. 3a).

We next evaluated the DNA damage response (p-H2AX and p-Chk2) in Tax inducible cell lines, with or without treatment with an iNOS inhibitor. We previously showed that induction of Tax in JPX9 cells was associated with an increase in the DNA damage response [26]. In this context, the inhibition of enzymatic activity by iNOS inhibitor, 1400 W, was associated with a reduction in the DNA damage response (Fig. 3b). We observed a decrease of p-Chk2 and p-H2AX in the TetOn-Tax cells treated with 1400 W (Fig. 3b). Cells treated with iNOS inhibitor expressed the same level of iNOS protein (Fig. 3c), but NO production was decreased (Fig. 3c). Thus, NO production correlated with the DNA damage response.

The role of Tax in maintaining the ATL phenotype remains an elusive question because of the existence of Tax-negative ATL cell lines [39, 40]. We wanted to determine whether the expression of Tax in ATL is a prerequisite for iNOS expression. The measurement by real time PCR of iNOS mRNA expression in three Tax-negative ATL cell lines, MT1, ED4505- and TL-Oml1 showed no expression of iNOS mRNA when compared to negative control CD4^+^ and Jurkat cells (Fig. 3d). Surprisingly, the expression of IκBα, which is a target gene of NF-κB transcription factors, was not detected in Tax-negative ATL cell lines and control CD4^+^ and Jurkat cells, but was positive in MT4 and HuT102, indicating that NF-κB activation is required for induction of iNOS expression (Fig. 3e). It has been previously shown that activation of CD4^+^ cells and macrophages results in the expression of iNOS [41, 42]. Our experiments showed that the combination of LPS with IFNγ or TNFα with IFNγ induces NO production in control CEM cells as well as in THP1 differentiated cells but not in HTLV-1 infected MT4 cells because they were already saturated (data not shown).

### Tax activates iNOS expression through the NF-κB pathway

**Fig. 4:**
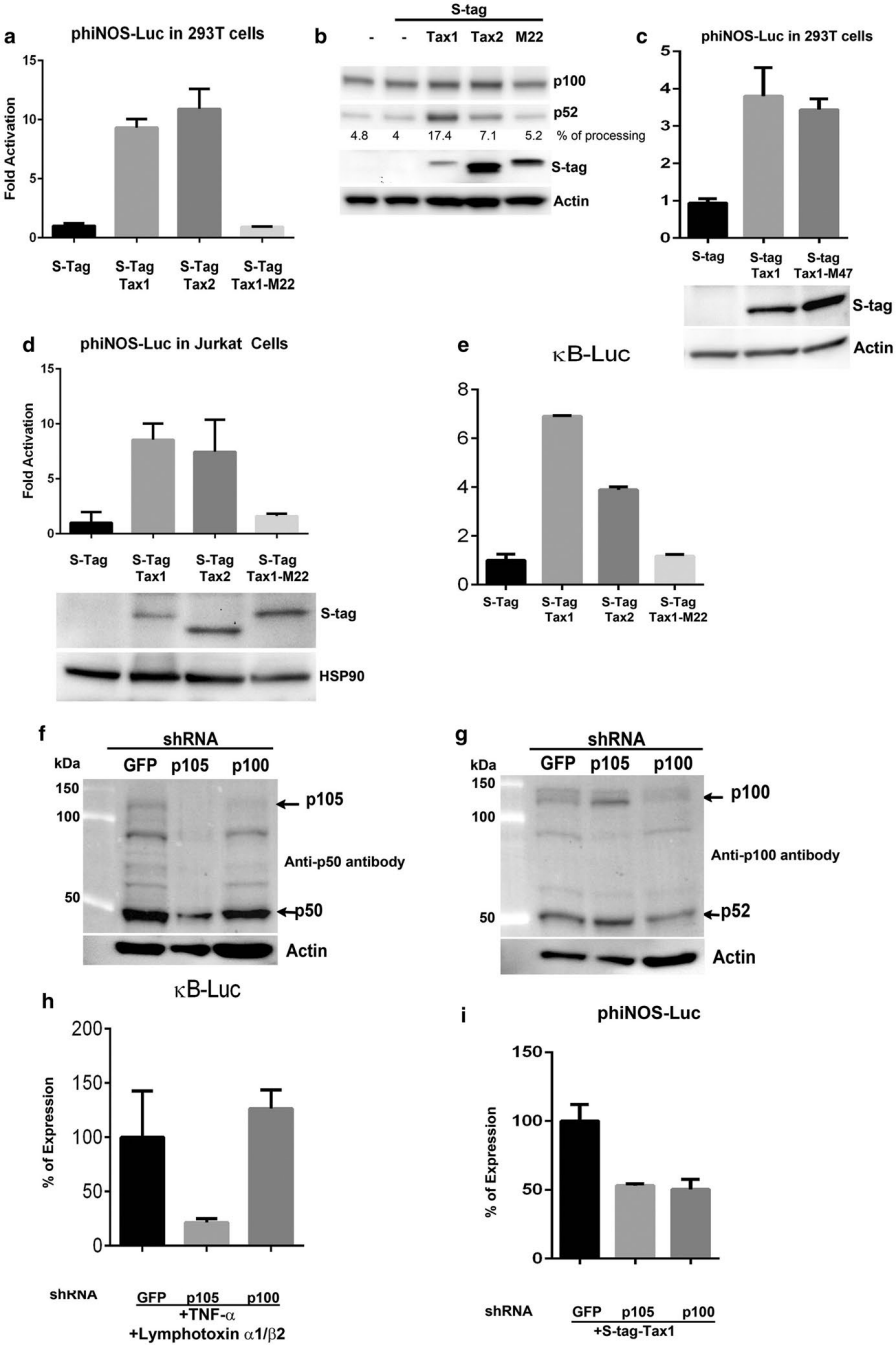
Tax activates iNOS expression through the NF-κB pathway. **a** A functional test showing the direct effect of Tax on iNOS expression. 293T cells were cotransfected with phiNOS-Luc along with vectors expressing S-tag control, S-tag Tax1, S-tag Tax2 or S-tag Tax1 M22 mutant. The transfection also included TK-RL to serve as a control of transfection. 48 h later, the luciferase activities were measured in the cell extracts. The histograms represent the average of three different experiments with the indicated standard deviation. **b** Western blot analysis showing that Tax1 but not Tax1 M22 protein analyzed in (**a**) was functional and able to process the p100 NF-κB2 protein to the p52 subunit using a specific anti-p100 antibody. **c** Similar transfection as in (**a**) was carried out on 293T cells with phiNOS-Luc and the vectors expressing S-tag control, S-tag Tax1, or S-tag Tax1 M47 mutant. **d** The effect of Tax on iNOS expression was also carried out in T cells (Jurkat), the physiological host of HTLV-1 infection, showing similar activation as shown in (**a**). **e** In parallel, 293T cells were co-transfected with κB-Luc reporter plasmid along with the same set of vectors used in (**a**) to show that Tax1, Tax2 but not Tax1M22 were able to activate the classical NF-κB pathway. **f**, **g** Stable Hela cells that express specific shRNA directed against mRNA of p105 and p100 were analyzed by Western blot using respectively specific anti p50 and anti p100 antibodies. **h** The same Hela cells were co-transfected either by κB-Luc reporter plasmid and induced by TNFα (1 ng/ml) and lymphotoxin α1/β2 (1 ng/ml) to induce the classical and alternative NF-κB pathways or **i** by phiNOS-Luc plasmid with the Tax-expressing vector to activate both NF-κB pathways. The expression of κB-Luc was inhibited with the depletion of p105. However, phiNOS-Luc expression was inhibited when either p105 or p100 were depleted.

We next explored the mechanism of Tax activation of iNOS expression in HTLV-1 infected cells. Since there is considerable literature on Tax activation of NF-κB, and NF-κB activation of iNOS [43–46], we first assessed the role of the NF-κB pathways in Tax activation of iNOS. Two pathways drive NF-κB induced transactivation. The p50/RelA and p52/RelB dimers are primary components of the classical and the alternative NF-κB pathways, respectively. To determine which NF-κB pathway is involved in iNOS activation, we used a reporter construct in which the expression of a luciferase reporter gene is driven by the human iNOS-promoter (phiNOS-Luc) [47]. We co-transfected 293T cells with phiNOS-Luc and with vectors expressing S-tag-Tax1 of HTLV-1 or S-tag-Tax2 of the related virus, HTLV-2. While Tax1 activates both NF-κB pathways, Tax2 exclusively activates the classical NF-κB pathway [48]. The luciferase activities measured after 48 h of transfection showed that both Tax1 and Tax2 were able to activate the iNOS promoter when compared to those measured in the control, transfected cells, suggesting that the classical NF-κB pathway is sufficient to activate iNOS expression (Fig. 4a). Expression of the S-tag-Tax1 M22 mutant, which is completely defective in NF-κB activation, showed no activity on the iNOS promoter, suggesting that NF-κB activation induces iNOS expression in HTLV-1 infected cells (Fig. 4a). A Western blot analysis was performed on the same cell extracts, showing that S-tag-Tax1, S-tag-Tax2, and S-tag-Tax1 M22 were expressed in the transfected cells (Fig. 4b). The Western blot in Fig. 4b also shows specific processing to p52 of the p100 alternative NF-κB subunit in cells-expressing Tax1, confirming the functional activation of the alternative NF-κB pathway by Tax1 but not Tax2 and Tax1 M22. In addition to NF-κB activation, Tax1 is known to transcriptionally activate HTLV-1 viral expression through the ATF-CREB pathway. To determine if the ATF/CREB pathway is critical in iNOS activation by Tax, we co-transfected 293T cells with phiNOS-Luc and with vectors expressing S-tag-Tax1 or S-tag-Tax1 M47 mutant, which is completely defective in the ATF/CREB activation (Fig. 4c). The luciferase activities showed that the ability to activate the expression of iNOS by Tax1 M47 was similar to that induced by Tax1 wild type, indicating that ATF/CREB was not involved in iNOS activation. The activation effect of iNOS promoter by Tax proteins was also confirmed by co-transfection of the same Tax expressing vectors with phiNOS-Luc reporter plasmid in Jurkat cells, indicating similar activation in T cells, the physiological host of HTLV-1 infection (Fig. 4d). In addition, the expression of Tax1, Tax2 and Tax1 M22 in cells-cotransfected with κB-Luc reporter plasmid, a promoter responsive to the classical NF-κB pathway, showed that Tax1 and Tax2 but not Tax1 M22 were able to activate the expression of κB-Luc, indicating that both wild type proteins were functionally active (Fig. 4e).

For further investigation of the specificity of iNOS activation, we made stable Hela cell lines that constitutively express either control shRNA or specifically targeted shRNA, to deplete the expression of p105 or p100 of the classical and alternative NF-κB pathways, respectively. The shRNAs were successful in reducing the expression of p105 and p100, as well as the processed p50 and p52 products, respectively (Fig. 4f, g). Transfection of these Hela cells with the κB-Luc reporter plasmid showed that κB-Luc activity is exclusively decreased when p105 was depleted, confirming that κB is activated by the classical pathway (Fig. 4h). Transfection of the same cells with phiNOS-Luc reporter plasmid showed that shRNA to p105 or p100 decrease iNOS activation, indicating that both NF-κB pathways contribute to iNOS activation (Fig. 4I).

### Tax-mediated iNOS expression also requires activation of the JAK/STAT pathway

**Fig. 5:**
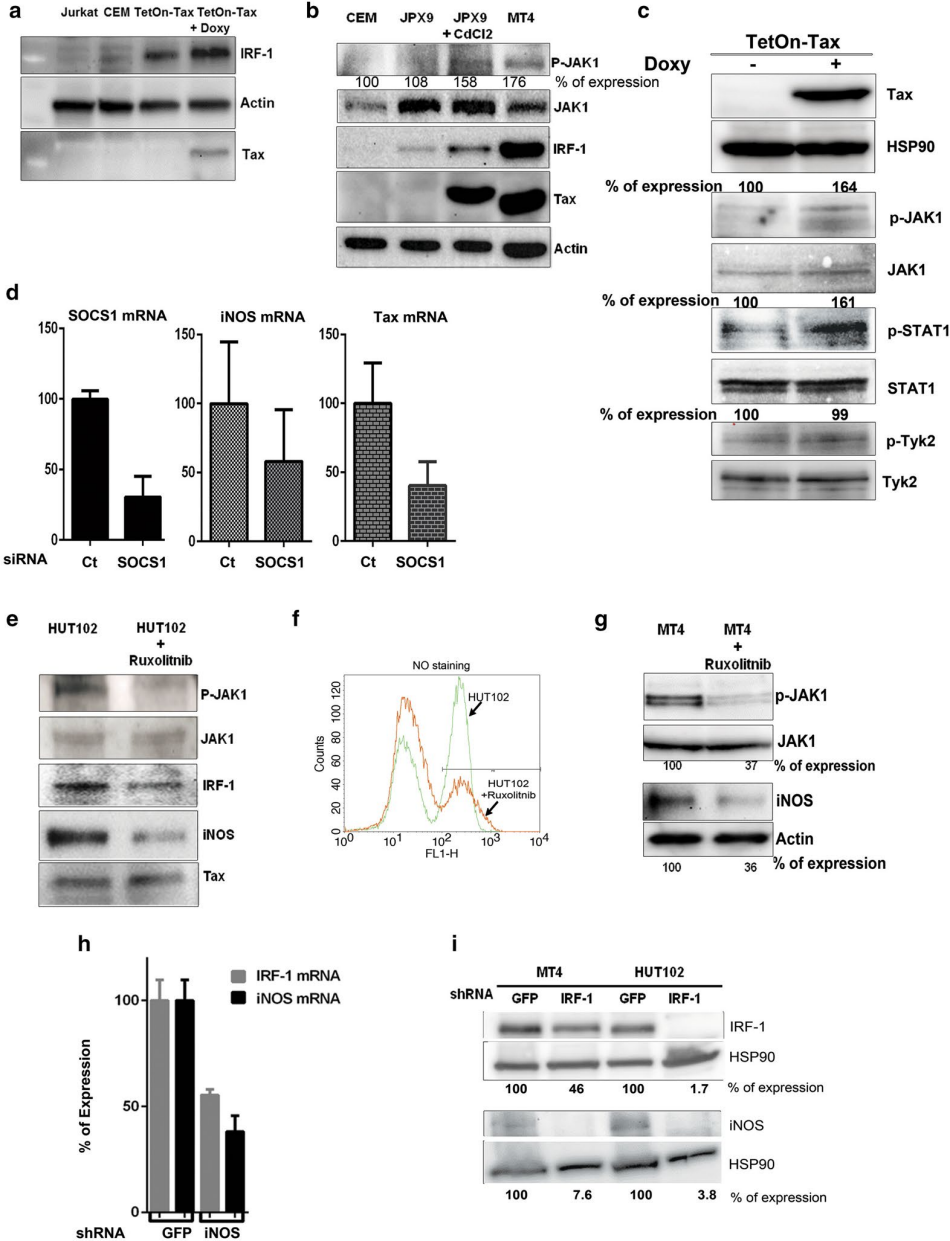
iNOS expression requires the activation of the JAK/STAT pathway by Tax. **a** iNOS expression correlates with IRF-1 expression in Tax expressing cells but not in Jurkat or CEM control cells. **b** Western blot analysis of key proteins in the JAK/STAT pathway, including JAK1, p-JAK1, and IRF1 in control CEM, non-induced JPX9, Tax-induced JPX9 (48 h with 40 μM CdCl_2_) and MT4 cells. Tax expression was also measured to show the specificity of expression in the HTLV-1 infected cell line and in the Tax inducible cell line. **c** TetOn Tax Jurkat cells were induced with doxycycline for 48 h and a Western blot analysis showing an increase of p-JAK1, p-STAT1 but not p-Tyk2 when Tax was induced. The percentage of expression was indicated on the top of each blot. **d** Real time measurement of SOCS1, iNOS and Tax mRNA in MT4 cells transfected with either control siRNA or specific siRNA directed against SOCS1 mRNA. **e**, **f**, **g** The expression of iNOS protein and NO production were reduced in HTLV-1 transformed cells lines (HuT102 and MT4) treated 48 h with Ruxolitinib (7 nM), a JAK1 and JAK2 inhibitor, showing the specificity of the JAK/STAT pathway in iNOS expression. The percentage of expression of p-JAK1 and iNOS are shown below each panel of the figure. **h** MT4 cells transfected with control siRNA or with siRNA specifically directed against IRF-1 mRNA. Total RNA was extracted, and IRF-1 and iNOS mRNA was quantified by real time PCR. **i** Stable MT4 and HUT102 cell lines that express shRNA directed against control mRNA (GFP) or against IRF-1 mRNA were also tested for IRF-1 and iNOS expression by Western blot. The percent of expression for IRF-1 and iNOS are shown below each panel. Both experiments show a correlation between IRF-1 and iNOS expression.

In addition to the NF-κB transcription factors, IFNγ was described as a key cytokine involved in iNOS expression. IFNγ is a type II interferon that activates the JAK/STAT pathway, a major signaling pathway involved in the regulation of the immune response. JAK/STAT induces an ensemble of interferon-stimulated genes including IRF-1, which has been shown to cooperate with NF-κB transcription factors to induce the expression of iNOS in melanomas [49]. We first checked the expression of IRF-1 in different cell lines, including control Jurkat, CEM, non-induced, and induced TetOn-Tax Jurkat cells. Interestingly, IRF-1 was induced when Tax expression was induced by doxycycline (Fig. 5a). Detection of lower levels of IRF-1 in TetOn-Tax Jurkat cells not treated with doxycycline, is likely due to leakiness of this cell line.

We next compared the activation of the JAK/STAT pathway in control CEM, non-induced JPX9, CdCl_2_-induced JPX9, and MT4 cells. Only Tax expressing cells, demonstrated an increase of p-JAK1 and IRF-1, indicating an activation of the JAK/STAT pathway (Fig. 5b) with Tax expression. We also observed a specific increase of p-JAK1 and p-STAT1 but not p-Tyk2 (activated only by type I interferon) in induced TetOn-Tax Jurkat cells suggesting a specific type II interferon activation of JAK/STAT (Fig. 5c). Although a general interferon response is induced after viral infection, HTLV-1 has evolved strategies to neutralize the interferon response. It has recently been shown that SOCS1 protein (Suppressor of cytokine signaling 1) is specifically induced by Tax to inhibit the interferon response [50]. SOCS1 is also induced by the interferon signaling pathways, but the mechanism and factors involved in its activation were not completely elucidated. To determine if SOCS1 induces a negative effect on iNOS expression, we transfected MT4 cells with either a control siRNA or a specific siRNA directed against SOCS1 mRNA and measured the mRNA pool of both SOCS1 and iNOS. The siRNA successfully inhibited more than 70% of SOCS1 expression. However, iNOS expression in MT4 cells transfected with SOCS1 siRNA was slightly inhibited and was also associated with Tax mRNA inhibition, which is likely due interferon-mediated restriction of viral expression. These results suggested that SOCS1 had no effect on iNOS expression in HTLV-1 infected cells (Fig. 5d).

Treatment of HTLV-1 transformed cell lines, HuT102 and MT4, with Ruxolitinib, a specific inhibitor of JAK1 and JAK2, caused a decrease of both iNOS protein expression and NO production (Fig. 5e–g). To confirm that the increase in IRF-1 expression is leading to iNOS activation, we measured the expression of IRF-1 and iNOS mRNA in MT4 cells transfected either with a control siRNA or with a specific siRNA directed against IRF-1 mRNA. The approximately 50% decrease of IRF-1 mRNA was associated with a similar decrease in iNOS mRNA expression (Fig. 5h). We also established MT4 and HUT102 cells that stably expressed specific shRNA directed against IRF-1 mRNA. As expected, the depletion of IRF-1 expression correlated with a decrease in iNOS expression, indicating that IRF-1 is one of the transcription factors involved in iNOS expression (Fig. 5i). Whether Tax directly activates the JAK/STAT pathway is still under investigation. However, data previously reported indicate that JAK/STAT might be induced at a later stage of infection by cytokines induced by Tax through the NF-κB pathway. Taken together, our results suggest that the NF-κB pathway has a central role in Tax-mediated iNOS expression in HTLV-1 infected cells.

## Discussion

ATLL is an aggressive fatal malignancy of CD4^+^ T-cells infected by HTLV-1 [51]. HTLV-1 induced leukemia/lymphoma, like most cancers, is characterized by three major hallmarks: activation of proliferation, inhibition of tumor suppressors, and genetic instability. While in other cancers, these hallmarks can be established by driver mutations, HTLV-1 induced tumorigenesis is driven by the expression of the viral oncogene, HTLV-1 Tax, which induces a potent inflammatory response through the NF-κB pathway [52, 53]. Inflammatory mediators have been shown to contribute to the proliferation of pre-neoplastic cells, but it is still largely unknown whether or not they contribute to the accumulation of genomic damage, a driving force for tumorigenesis [30].

A role of Tax in inducing genomic damage has been under active investigation during the last decade. Tax targets proteins involved in DNA replication [54, 55], DNA damage and repair [26, 56], centrosome duplication [57], and cell cycle and cell division checkpoints [58–60]. The long period of latency and the low percentage of infected cells which are ultimately transformed suggest that Tax is able to induce random mutations. In this context, Tax has been shown to induce DNA double strand breaks (DSB) [26], the most serious form of genomic damage. Since the NF-κB pathway is a critical determinant in Tax-mediated tumorigenesis [52], we wanted to delineate how inflammatory mediators contribute to the accumulation of genomic damage. Here we show that in HTLV-1 infected cells, Tax activates the expression of iNOS, the enzyme that catalyzes the production of NO in T-cells, macrophages and neutrophils. Moreover, we demonstrated that Tax induces DSBs through the induction of NO by the classical NF-κB and JAK/STAT pathways, which are implicated in the proliferation of HTLV-1 infected T-lymphocytes [61]. The activation of iNOS expression is at the intersection between these two pathways.

The central hypothesis of the study is that chronic inflammation induced by the NF-κB pathway induces DNA damage through activation of iNOS. Although several transcription factors were reported to activate iNOS expression through inflammation-mediated activation [62], Tax activation of NO production through the NF-kB pathway provides a novel insight into the mechanisms regulating genomic instability and leading to ATLL. There is no doubt that Tax is required in the initials events, including genomic defects, which drive the leukemogenic process in HTLV-1 infected cells.

Recent studies showed that other HTLV-1 viral proteins [63] or other mechanisms [31] might be involved in NF-kB activation. Whether NF-kB constitutive activation in ATL cells requires the maintenance of Tax expression is still under intensive investigation. Nevertheless, HTLV-1 Tax remains a potent driver of NF-kB activation in the HTLV-1 infection model. On the other hand, the common concept that Tax is not detected in ATL cell lines does not mean that the protein is not expressed. Tax mRNA expression in ATL cell lines has been reported in multiple studies, and the NF-κB pathway might be activated with very low or intermittent levels of Tax which may be undetectable unless highly sensitive techniques are employed.

In HTLV1-mediated leukemogenesis as in other cancers, the driving events are associated with inactivation of tumor suppressors [64], which favors the accumulation of more cellular defects and contributes to the development of cancer. If NO is produced at low concentrations in macrophages or activated T-cells, it promotes tumor cell proliferation, migration, invasion, and an environment resistant to apoptosis [19]. Previous studies showed that NO produced by the tumor microenvironment is involved in cancer development. However, the effects of cell intrinsic mechanisms of tumor promotion by NO have not been thoroughly studied. In particular, it is unclear how NO induces mutations and progressive genetic alterations via increased oxidative and nitrosative stress.

Tax is responsible for a wide range of oncogenic events associated with HTLV-1 infection [5]. It is still unclear whether DSBs are induced by Tax through the stimulation of NO production alone. Indeed, 30–40% of the DSBs, typically found in Tax expressing cells, were still present after iNOS inhibition. This observation suggests that Tax may be able to induce DSBs through another mechanism, or that inhibition of iNOS is not complete. In this context, Tax may induce stalling of the DNA replication fork by targeting PCNA or topoisomerase I, two critical proteins implicated in DNA replication [65, 66]. Tax has also been shown to increase the production of other reactive oxygen species, which are known to induce DNA damage [67]. Organic hydroperoxides, present in cancer cells could also contribute to the observed phenotype. The effects of reactive oxygen species, other than NO, need to be investigated in future studies. To further delineate the mechanism of NO-mediated DSBs, future experiments will also be required to investigate the dynamic relationship between NO release and DSBs and test whether NO affects DNA by direct chemical interaction or through another mechanism.

A recent publication showed that Tax attenuates DNA damage responses by inhibiting ATM through activation of phosphatase WIP1 [68]. In the study, the authors used UV-induced DNA damage response to investigate Tax activity. Given that Tax can be involved in both induction of DNA damage and inhibition of DNA repair, the present study aimed to investigate a mechanism by which Tax induces DSBs through inflammatory mediators in the absence of external DNA damage.

Although we found that NO induces DSBs and promotes persistent genomic instability, this model only explains how HTLV-1 infected cells may acquire the oncogenic phenotype during infection. However, it does not provide an explanation for a requirement for sustained NO production in HTLV-1 transformed cell lines, which have already acquired the transformed phenotype. Thus, NO might be required for oncogenic mechanisms other than the induction of DNA damage. NO is known to increase the nitrosylation of proteins: this posttranslational modification may lead to either inactivation or activation of function. Therefore, nitrosylation of key proteins, implicated in T-lymphocyte proliferation, should be tested in order to further delineate the role of NO in transformed cell lines.

## Conclusion

Genetic instability and DSBs in particular are believed to be a driving force for tumorigenesis. Here, we found that the release of nitric oxide was associated with an increase of DNA damage in HTLV-1 infection model, suggesting that NO might be an important source of genetic instability in HTLV-induced leukemia. Determination of the impact of NO on tumor formation in an ATLL model will help in the development of alternative strategies for therapeutic applications of ATLL.

## Methods

### Cell lines, ATL patients, culture and drugs

All cell lines used in this study were obtained from the ATCC (American Type Culture Collection). HTLV-I transformed human T-cell lines MT2, MT4, and HuT102, control T-cell lines CEM and Jurkat, and Tax inducible transformed cell lines were all cultured in RPMI 1640 (Sigma) with 10% fetal bovine serum (Sigma), supplemented with 2 mM glutamine, penicillin (100 U/ml), streptomycin (0.1 mg/ml), and amphotericin B (0.25 µg/ml). JPX9 is a Jurkat subclone generated by stable transfection of an inducible Tax expression vector driven by the metallothionein promoter (made by Prof Sugamura, Tohoku University, Sendai, Japan) [37]. TetOn-Tax is also a Jurkat subclone generated by introduction of an inducible TetOn Tax expression vector (made by Prof W C Greene, University of California, San Francisco, USA) [38]. Peripheral blood mononuclear cells (PBMC) were isolated from healthy donors, purified on Ficoll, and CD4^+^ T-cells were separated using Easy Step Human CD4^+^ T Cell negative selection (STEMCELL Technologies). PBMCs from three ATLL patients with acute ATLL were also collected and frozen in 2010 under the patient’s consent. Details about ATLL patients’ samples are given in Table 1. 293T cells were maintained in Dulbecco’s modified Eagle medium, DMEM, supplemented with the same reagents as RPMI as well as 1 mM sodium pyruvate. Cells were maintained at 37°C in a humidified incubator with 5% CO2 concentration. 1400 W, a selective inhibitor of iNOS was purchased from Cayman chemicals and was used at the concentration of 7 nM for the time indicated in the figure legends. cPTIO ((2-(4-Carboxyphenyl)-4,4,5,5-tetramethylimidazoline-1-oxyl-3-oxide), which is an NO scavenger, was used at a concentration of 100 μM (Santa Cruz Biotechnologies).

### Co-culture of MT2 and PBMC

Lethally irradiated MT2 cells (10,000 cGy) were co-cultured with fresh PBMCs from healthy donors (ratio 1–10, MT2/PBMC). The PBMCs were cultured in complete RPMI (10% FBS) with PHA 1 μg/ml and 50 units/ml IL-2. The freshly infected PBMC were monitored side by side with irradiated MT2, non-irradiated MT2 cells, and uninfected PBMCs for 2–3 weeks, and RNA was extracted for real time measurements of iNOS and GAPDH mRNA.

### Plasmids, lentiviral vectors and transfection methods

The vector TetOn-pLKO1 puro, used to induce the expression of iNOS shRNA, was purchased from Adgene (plasmid 21915) [69]. The sequences of oligos cloned into AgeI and EcoRI sites in the vector to create the respective shRNAs are listed in Table 2. Lentiviral particles expressing shRNAs were prepared by transfecting 293T cells with TetOn-pLKO1-shRNA, HIV-gag-pol (10 µg) and VSV-G (10 µg) expressing plasmids using calcium phosphate. Supernatant was collected at 24, 48 and 72 h, and lentiviral particles were concentrated by ultracentrifugation. To deplete the expression of IRF-1, pLKO-1 puro vectors stably expressing five different shRNA that target IRF-1 mRNA were provided by the Genome Institute at Washington University. The target sequences of these shRNAs are given in Table 2. The determination of virus titer by the infection of control cells gives the number of viruses required to infect target cells at an MOI of 1. Lentiviral particles were used to transduce MT4 cells in the presence of polybrene 12 μg/ml, and cells were selected in puromycin (2 μg/ml) starting 48 h later. The S-Tag Tax-1 plasmid was a generous gift from O. John Semmes [70]. The S-Tag Tax-1 M22 expression plasmid contains the Tax M22 mutation defective in NFκB activation [71]. S-Tag Tax-2 plasmid was constructed by amplifying the open reading frame of Tax from BC20.2 [72] and was inserted into pTriEx™-4 Neo vector (Novagene, Madison, WI, USA) in-frame with the amino-terminal S-tag. The M47 mutation was amplified with internal primers from pACH M47 and was swapped with Tax1 in S-Tag Tax1 to create S-Tag Tax1 M47. Plasmid structures were confirmed via sequence analysis. The plasmid phiNOS-Luc expressing the luciferase gene under the control of hiNOS promoter was a gift from Dr. David A. Geller (Director, UPMC Liver Cancer Center, University of Pittsburgh) [47]. κB-Luc expressing the luciferase gene under the control of 5 κB responsive elements was a gift from Dr. D Piwnica-Worms [73]. The plasmid pRL expressing the renilla luciferace under the promoter of thymidine kinase was used for transfection efficiency and for normalizing the luciferase activities (Promega). The transfection of 293T and Hela cells was performed using TransIT reagent based on the manufacture’s recommendations (Mirus Bio), and the luciferase activities were calculated using a dual luciferase solution. Jurkat and MT4 cells were transfected by electroporation using the BTX electroporation system 600. siRNA control or that directed against IRF-1 or SOCS1 mRNA were purchased from Santa Cruz Biotechnology (SC-35706, SC-40997).

**Table 2:** Sequences of shRNA used to deplete the corresponding targets, and primers used for real time PCR

pLKO-1 shRNA IRF-1 target sequences	
IRF-1	CGTGTGGATCTTGCCACATTT
IRF-1	CCTCTGTCTATGGAGACTTTA
IRF-1	GCAGATTAATTCCAACCAAAT
IRF-1	AGATGCTAAGAGCAAGGCCAA
IRF-1	GCGTGTCTTCACAGATCTGAA
TetOn-pLKO-1 shRNA	
hiNOS	Forward: 5′-CCGGAACATTGCTGTGCTCCATAGTCTCGAGACTATGGAGCACAGCAATGTTTTTTG-3'
hiNOS	Reverse: 5′-AATTCAAAAAAACATTGCTGTGCTCCATAGTCTCGAGACTATGGAGCACAGCAATGTT-3'
GFP	Forward: 5′-CCGGCAGCCACAACGTCTATATCATCTCGAGATGATATAGACGTTGTGGCTGTTTTTG-3'
GFP	Reverse: 5′-AATTCAAAAACAGCCACAACGTCTATATCATCTCGAGATGATATAGACGTTGTGGCTG-3'
Real time PCR primers	
hGAPDH	Forward: 5′-GTGAAGGTCGGAGTCAACGG-3′
	Reverse: 5′-AGTGATGGCATGGACTGTGG-3′
hiNOS	Forward: 5′-ACAACAAATTCAGGTACGCTGTG-3′
	Reverse: 5′-TCTGATCAATGTCATGAGCAAAGG-3′
IκBα	Forward: 5′-TGTCTACACTTAGCCTCTATC-3′
	Reverse: 5′-TCTGTGAACTCCGTGAACTC-3′
IRF-1	Forward: 5′-GAACTCCCTGCCAGATATCGAG-3′
	Reverse: 5′-TGCTCTTAGCATCTCGGCTGG-3′
SOCS1	Forward: 5′-TTTTTCGCCCTTAGCGTGA-3′
	Reverse: 5′-AGCAGCTCGAAGAGGCAGTC-3′
Tax	Forward: 5′-CGGATACCCAGTCTACGTGT-3′
	Reverse: 5′-GAGCCGATAACGCGTCCATCGATG-3′

### Real time RT-PCR

The expression of human iNOS, IκBα, IRF-1, SOCS1 and HTLV-1 Tax was quantified by real time PCR using Biorad iCycler. Briefly, the total mRNA of HTLV-1 positive and negative cell lines was extracted by Trizol, DNase-I treated and reverse-transcribed using the High capacity RNA to cDNA kit (Applied Bioscience) as recommended by the manufacturer. Quantitative reverse transcriptase PCR (40 cycles) was performed in a total of 20 μl containing the iQ Sybr Green supermix reagent (Bio-rad), deionized water, primers at 0.5 μM each and cDNA as recommended by the manufacturer. Human GAPDH was used as an internal amplification control. Relative expression was determined by the delta delta C_t_ method. The fold change = $2^{{ - \Delta \Delta C_{T} }}$.

### Western blot

Cell extracts were prepared using RIPA lysis buffer (50 mM Tris–HCl pH 7.5, 150 mM NaCl, 1% NP-40, 0.1% SDS, 0.5% DOC and protease inhibitor cocktail (Roche). Proteins were quantified by Bradford assay and separated by SDS PAGE, transferred to PVDF membranes and detected using the standard protocol for Western Blot. Primary antibodies used in this study were directed against the following proteins: iNOS SC-651 (Santa Cruz), Actin (SC-1615), and Tax mouse monoclonal (NIH AIDS Reagent Program, HTLV-I Tax Hybridoma (168B17), p-JAK1 (SC-16773), JAK (SC-277), p-STAT1 (SC-7988), STAT1 (SC-592), IRF1 (SC-497), p-Chk2 (SC-16279), p-H2AX (Biolegend 613402), Chk2 (SC-2662p), H2AX (Biolegend 613301) p100/p52 (Millipore 05-361), p50 (SC-1190) and S-Tag (Millipore MAC 112). Signals were detected using the ChemiDoc XRC from Bio-Rad.

### Immunocytofluorescence staining and microscopy

T-cell lines were cytospun onto slides at 800 rpm for 5 min. The cells were then fixed in 3.7% paraformaldehyde (PFA) for 15 min at RT, washed with PBS, permeabilized on ice for 5 min with 0.5% Triton X-100, and blocked for 1 h in PBS with 0.5% gelatin and 0.25% bovine serum albumin at room temperature. Slides were next incubated with anti-iNOS and anti-p-H2AX antibodies at 1/200 in PBS for 2 h, washed three times in PBS-0.2% gelatin for 10 min each time, and incubated with Alexa Fluor 488-conjugated goat anti-rabbit secondary antibody and Alexa Fluor 596-conjugated goat anti-mouse secondary antibody (Molecular Probes, Invitrogen) in PBS-0.2% gelatin for 1 h at room temperature. Cells were washed three times in PBS-0.2% gelatin for 10 min each time and mounted using DABCO mounting medium (2.5% DABCO from Sigma, 200 mM Tris–HCl pH 8.6 and 90% glycerol). Fluorescent images were captured using a Nikon Eclipse Tis epifluorescence microscope and the NIS elements software (Nikon). The images were collected by using the objectives 20X and 40X, and the number of DNA Double-Strand Breaks (DSB) foci and the quantification of fluorescence intensity was performed using the object count module in NIS elements.

### Comet assay

Comet Assay is a single cell electrophoresis technique that provides a simple and effective method for evaluating DNA damage in cells. NO and its reactive products induce DNA modifications that start as single-strand breaks, which result in DNA double-strand breaks. We performed the Comet Assay alkaline method that detects both single-strand and double-strand breaks based on the manufacturer’s protocol (Trevigen).

### Flow cytometry

Intracellular staining of NO pools was performed according to the manufacturer’s instructions (Cayman) with 4,5-Diaminofluorescein Di-acetate (DAF-2DA). Annexin V/Propidium iodide kit (BD Biosciences) was used to measure apoptosis in cells treated with iNOS inhibitor, or cells expressing shRNA according to the manufacturer’s instructions. Cells were analyzed by FACS Caliber.
